# The four-minute approach revisited: accelerating MRI-based multi-factorial age estimation

**DOI:** 10.1007/s00414-019-02231-w

**Published:** 2019-12-19

**Authors:** Bernhard Neumayer, Andreas Lesch, Franz Thaler, Thomas Widek, Sebastian Tschauner, Jannick De Tobel, Thomas Ehammer, Barbara Kirnbauer, Julian Boldt, Mayonne van Wijk, Rudolf Stollberger, Martin Urschler

**Affiliations:** 1 Ludwig Boltzmann Institute for Clinical Forensic Imaging, Universitätsplatz 4, 8010 Graz, Austria; 2 BioTechMed-Graz, Graz, Austria; 3 Institute of Medical Engineering, Graz University of Technology, Stremayrgasse 16, 8010 Graz, Austria; 4 Institute of Computer Graphics and Vision, Graz University of Technology, Inffeldgasse 16, 8010 Graz, Austria; 5 Division of Pediatric Radiology, Department of Radiology, Medical University of Graz, Auenbruggerplatz 34, 8036 Graz, Austria; 6 Department of Diagnostic Sciences – Radiology, Ghent University, C. Heymanslaan 10, 9000 Ghent, Belgium; 7 Department of Imaging and Pathology - Forensic Odontology, KU Leuven, Kapucijnenvoer 7 blok a bus 7001, 3000 Leuven, Belgium; 8 Division of Oral Surgery and Orthodontics, Department of Dental Medicine and Oral Health, Medical University Graz, Billrothgasse 4, 8010 Graz, Austria; 9 Department of Prosthodontics, University of Würzburg, Pleicherwall 2, 97070 Würzburg, Germany; 10 Netherlands Forensic Institute, Laan van Ypenburg 6, 2497 GB The Hague, The Netherlands; 11 School of Computer Science, University of Auckland, 23 Symonds Street, Auckland, New Zealand

**Keywords:** Reproducibility of results, Age determination by skeleton, Age determination by teeth, Imaging, Three-dimensional, Neural network models

## Abstract

**Objectives:**

This feasibility study aimed to investigate the reliability of multi-factorial age estimation based on MR data of the hand, wisdom teeth and the clavicles with reduced acquisition time.

**Methods:**

The raw MR data of 34 volunteers—acquired on a 3T system and using acquisition times (*T*_A_) of 3:46 min (hand), 5:29 min (clavicles) and 10:46 min (teeth)—were retrospectively undersampled applying the commercially available CAIPIRINHA technique. Automatic and radiological age estimation methods were applied to the original image data as well as undersampled data to investigate the reliability of age estimates with decreasing acquisition time. Reliability was investigated determining standard deviation (SSD) and mean (MSD) of signed differences, intra-class correlation (ICC) and by performing Bland-Altman analysis.

**Results:**

Automatic age estimation generally showed very high reliability (SSD < 0.90 years) even for very short acquisition times (SSD ≈ 0.20 years for a total *T*_A_ of 4 min). Radiological age estimation provided highly reliable results for images of the hand (ICC ≥ 0.96) and the teeth (ICC ≥ 0.79) for short acquisition times (*T*_A_ = 16 s for the hand, *T*_A_ = 2:21 min for the teeth), imaging data of the clavicles allowed for moderate acceleration (*T*_A_ = 1:25 min, ICC ≥ 0.71).

**Conclusions:**

The results demonstrate that reliable multi-factorial age estimation based on MRI of the hand, wisdom teeth and the clavicles can be performed using images acquired with a total acquisition time of 4 min.

## Introduction

Age estimation in living individuals is important for clinical applications [1, 2], in legal or forensic investigations [3, 4] and sports [5, 6], but is prone to uncertainty caused by the variation of human development [7]. A living person’s chronological age is derived from their biological age, which is an active topic of current research [8]. Currently, particularly forensic age estimation receives wider attention due to the ongoing flow of individuals into and across the European Union, since it is legally necessary to determine whether individuals without valid identification documents, who claim to be minors, have reached the age of majority.

As recommended by the work group for forensic age diagnostics [4], imaging-based multi-factorial age estimation methods involve a radiograph of the hand [9, 10], a panoramic X-ray of the teeth [11] and computed tomography (CT) images of the clavicles [12]. The application of ionizing radiation associated with these imaging modalities prompted numerous studies to investigate magnetic resonance imaging (MRI) for its potential to replace the currently applied imaging techniques for the hand [13–15], teeth [16–18] and the clavicles [19–21], and to identify new age-relevant body regions [22–24].

Compared to CT and X-ray imaging, however, MRI generally requires considerably longer acquisition times. This leads to increased examination costs and reduced patient comfort and gives rise to potential errors due to motion artefacts when acquiring images of children or adolescents. Hillewig et al. were the first to address this problem with regard to forensic age estimation proposing a *four*-*minute approach* for MRI acquisitions of the clavicles [25] by comparing different MRI sequences and identifying the best compromise between acquisition time and image quality. Latest developments in MRI, however, allow to further reduce acquisition time by applying undersampling strategies. For age estimation, the first results have been reported by Terada et al. [26] and Neumayer et al. [27] for accelerated images of the hand and wrist. In the current work, we extend this approach to image data of the clavicles and the teeth, as these are the three anatomical structures required in the widely used multi-factorial age estimation scheme recommended by the work group for forensic age diagnostics [4] and have been proven to be most useful in majority age classification [28, 29].

For this purpose, we *retrospectively undersampled* MRI acquisitions of the left hand, the clavicles and the wisdom teeth and applied radiological and automatic age estimation methods to determine limits of acceleration that can be applied to MRI data without considerably influencing the outcome of the respective age estimation technique.

## Materials and methods

### Study design

The study was performed in accordance with the Declaration of Helsinki and was approved by the ethical committee of the local medical university. All eligible volunteering participants provided written informed consent; for underage participants, written consent from the parents was obtained.

### Subjects

For this feasibility study, 34 healthy male Caucasian volunteers between 13.37 and 24.05 years (mean 17.15 years, median 16.89 years, standard deviation 2.87 years) were recruited to acquire three-dimensional MR images of the left hand and wrist, the clavicles and the teeth. For one additional volunteer (19 years), whose image data was not used for retrospective undersampling, we additionally acquired images with undersampling factors 4, 2 and 6 for the hand, the clavicles and the teeth, respectively.

### MR acquisitions

MRI exams were performed using clinical 3T MR scanners (Skyra/Prisma, Siemens Healthineers, Erlangen, Germany).

Three fully sampled acquisitions formed the basis for our study:

•Hand: T_1_-weighted 3D VIBE, T_E_/T_R_/FA = 4.06 ms/14 ms/15°, field-of-view (FOV) = 129 mm × 230 mm, 2 averages, acquisition matrix = 129 × 230 and image matrix = 288 × 512, 72 slices, image resolution 0.45 mm × 0.45 mm × 0.90 mm, acquisition time *T*_A_ = 3:46 min.

•Clavicles: T_1_-weighted 3D VIBE, T_E_/T_R_/FA = 3.72 ms/9.77 ms/12°, FOV = 149 mm × 170 mm, 2 averages, acquisition matrix = 168 × 192 and image matrix = 224 × 256, 44 slices, resolution 0.90 mm × 0.90 mm × 0.90 mm, *T*_A_ = 5:29 min.

•Teeth: T_1_-weighted 3D TSE, T_E_/T_R_/FA = 12 ms/254 ms/150° (refocussing), TF = 4, FOV = 103 mm × 150 mm, acquisition matrix = 176 × 256 and image matrix = 352 × 512, 56 slices, image resolution 0.30 mm × 0.30 mm × 1 mm, *T*_A_ = 10:46 min.

For acquisitions of the hand and wrist, volunteers were placed in prone position with outstretched left arm and a sandbag placed on top of the hand to minimize movements and using a conventional 20-channel receive-only head-neck coil (Siemens Healthineers, Erlangen, Germany).

Images of the clavicles and the teeth were acquired in supine position, using a 4-channel neck coil (Siemens Healthineers, Erlangen, Germany) and an 8-channel multifunctional coil (CPC, Noras MRI products GmbH, Höchberg, Germany), respectively.

### Retrospective undersampling of MRI data

The principle of undersampled MRI exploits the redundancy of image information for acquisitions with multiple coil elements. This redundancy allows to acquire a smaller number of data lines than is required for a fully sampled data set. The missing information is then recovered by applying algorithms either based on parallel imaging [30, 31] or compressed sensing [32]. The resulting speed-up is referred to as the acceleration factor (AF), which is defined by the number of acquisition lines required for a fully sampled data set divided by the number of acquisition lines of the undersampled data set (e.g. acquiring only half of the data corresponds to an acceleration factor of AF = 2).

In contrast to standard image data provided by MRI scanners, MRI raw data—available at the scanner for a limited time after the scan due to its extensive storage requirements—includes the entire, unedited and non-combined data of each coil element. This allows removing data lines from a fully sampled raw data set *prior to image reconstruction*, which is equivalent to not collecting these lines during image acquisition. Therefore, retrospective undersampling of MRI raw data is a valid simulation of actual, undersampled acquisitions and additionally allows a comparison with the fully sampled data for the same subject in the same position. Since undersampling is performed retrospectively in this study, the prospective acquisition time of undersampled data will be termed *theoretical acquisition time* (*T*_A,th_) throughout this paper.

In this study, we generally followed the approach for images of the hand proposed in [27]: Retrospective undersampling of raw MRI data was performed by applying the commercially available CAIPIRINHA (controlled aliasing in parallel imaging results in higher acceleration) acquisition strategy [33] using the AVIONIC toolbox [34]. Coil sensitivities were estimated applying the ESPIRiT method using the BART toolbox [35] and image reconstruction was carried out using total generalized variation (TGV), which considers piecewise smooth intensity variations [36]. Only non-averaged data were undersampled; this reduced the required theoretical acquisition time by a factor of two, compared to the standard setting of performing two averages for acquisitions of the hand and wrist and the clavicles. For readability, images reconstructed from fully sampled MR data will be addressed as *original* images or data and images reconstructed from retrospectively undersampled data will be termed *undersampled* for the remainder of this paper.

Image data of all three body regions were undersampled according to Tables 1 and 2 for radiological and automatic age estimation, respectively. The applied acceleration factors for the hand were based on existing work, while for the clavicles and the teeth the degree of acceleration was chosen considering limiting factors for our method. For the analysis of the hand, radiologists were presented images undersampled with acceleration factors 4 and 8, based on the results of [27]. To keep the effort in reasonable bounds, radiologists and dentists were presented original and three undersampled data sets per volunteer for the clavicles and the teeth. The maximum AF for the clavicles was chosen to be 4 (the number of available channels); for the teeth maximum AF was set to 6 (slightly below the channel number due to coil arrangement). Automatic age estimation evaluated a larger set of undersampled image stacks up to an AF of 16 for the hand and an AF of 9 for the clavicles and the teeth. It has to be noted that undersampling strategies require an additional acquisition of a small number of calibration lines. Therefore, the actual speed-up will always be below the defined AF value; the actual acceleration factor is reflected in the resulting theoretical acquisition times in Tables 1 and 2.

**Table 1:** Acceleration factors applied for radiological age estimation. A value of AF = 1 designates original acquisition times. Theoretical acquisition times for acquisitions of the hand and clavicles are additionally halved by only using non-averaged data

	Hand			Clavicles				Teeth			
AF	1	4	8	1	2	3	4	1	2	4	6
*T*_A,th_ (s)	226	29	16	329	85	58	45	646	343	191	141

*AF* acceleration factor, *T*_A,th_ theoretical acquisition time

**Table 2:** Acceleration factors applied for automatic age estimation. A value of AF = 1 designates original acquisition times. Theoretical acquisition times for acquisitions of the hand and clavicles are additionally halved by only using non-averaged data

**Hand**									
*AF*	1	4	6	8	9	10	12	14	16
*T*_A,th_ (s)	226	29	20	16	14	13	11	10	9
**Clavicles**									
*AF*	1	2	3	4	6	8	9		
*T*_A,th_ (s)	329	85	58	45	32	25	23		
**Teeth**									
*AF*	1	2	3	4	6	8	9		
*T*_A,th_ (s)	646	343	242	191	141	115	107		

*AF* acceleration factor, *T*_A,th_ theoretical acquisition time

### Skeletal rating

Skeletal age was rated independently using two different approaches: application of (i) radiological methods by raters with the respective expertise and (ii) an automatic method based on deep convolutional neural network (DCNN) architectures for age estimation.

For images of the hand, radiologists applied the method proposed by Greulich and Pyle [9] (GP), which was originally based on radiographs but was recently verified for applicability [37] and reliability [27] when used for MR images. For the clavicles, nine different developmental stages were assigned as already performed on MR images in [19] and stages of teeth development were assessed as defined by Demirjian [11]. To avoid biased age estimates, MR images were anonymized and randomized irrespective of the acceleration factor. All raters were instructed to provide ratings only in clear cases, i.e. when an unambiguous assignment of a stage was possible. This further defined assessability of the data sets: the absence of a rating was tantamount with the data set being not assessable.

Given the anatomical differences between data sets, radiological assessment was performed by several raters to benefit from the specialisation of each evaluator. A paediatric radiologist with 6 years of experience in bone age estimation (R1) evaluated images of the hand. An oral and maxillofacial surgeon in training, with specific expertise in head and neck imaging and forensic odontology specially trained for the evaluation of the clavicles and with more than 7 years of experience in this field (R2) assessed images of the clavicles. A radiologist with more than 7 years of expertise in forensic applications (R3) evaluated images of the hand and the clavicles. A dentist with 10 years of experience in radiological evaluation of MRI data and 9 years of experience in age estimation (R4) and a specialist in oral surgery and oral radiology performing age estimations in the daily routine with 13 years of experience (R5) assessed images of the teeth. Due to the challenging aspects of MR images of the clavicles, a forensic anthropologist (R6) was appointed as a third evaluator for this data set, and raters R2 and R3 evaluated the original images a second time.

Automatic skeletal age estimation was performed using the fully automated method recently proposed by Štern et al. [28]. This method was evaluated on 322 data sets of subjects different to our cohort, but acquired with the same MRI protocol and provided a mean absolute error (MAE) of 1.01 ± 0.74 years (MAE ± standard deviation).

### Statistical analysis

Our focus in this study was on the reliability of multi-factorial age estimation with decreasing acquisition time instead of the absolute agreement with chronological age. For this purpose, we analysed the change introduced into the estimated age (automated), age category (hand) or developmental stage (clavicles and teeth) with increasing acceleration factor as proposed in [27]. As an estimator for this variation, we calculated the difference between the age/age stage estimated from original data (Age_*orig*_) from the age/age stage estimated from undersampled data (Age_*us*_):

$$ \varDelta \mathrm{Age}={\mathrm{Age}}_{us}-{\mathrm{Age}}_{orig} $$

For simplicity, ΔAge is used for both age differences and differences between estimated stages. The standard deviation of the signed differences (SSD) of ΔAge was used as a measure for the reliability of the age estimation, the mean of signed differences (MSD) served to identify potential systematic errors. Intra-class correlation (ICC) was calculated between age estimates based on original images and the estimates from undersampled data sets for each rater. Additionally, ICC and overall Bland-Altman mean (μ_BA_) and limits of agreement (LOA) between raters were determined.

### Best-performing combinations of undersampled data

To analyse the acceleration potential of MR acquisitions of each of the three body regions, all available data sets were combined in all valid compositions, i.e. one data set of each body region per volunteer in all combinations of available acceleration factors (see Table 2), to retrieve corresponding age estimates.

The reliability of all age estimates was analysed to identify the combinations that provide the best reliability while requiring the shortest possible *T*_A,th_. Besides reliability, agreement with chronological age was also investigated for selected combinations.

All statistical analyses were performed using MATLAB (R2017b, The MathWorks Inc., Natick, MA, USA).

## Results

### Available data

Two acquisitions of the hand were excluded from the evaluation due to strong artefacts (radiofrequency-based, motion) in the images. Additionally, MR raw data could not be obtained for one acquisition of the teeth. This ultimately resulted in 96 data sets of the hand, 136 data sets of the clavicles and 132 data sets of the teeth assessed for radiological age estimation.

### Image reconstruction and image quality

**Fig. 1:**
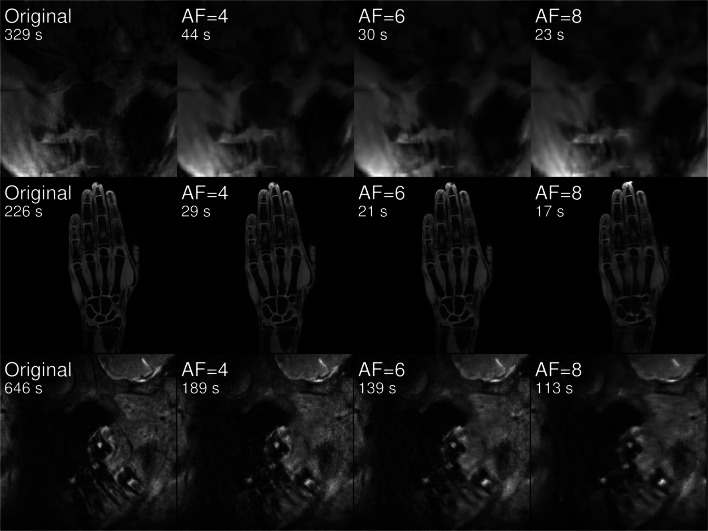
Exemplary original and undersampled images of all three body regions for one volunteer (13.8 years)

Figure 1 shows representative images of central slices of all three acquired data sets of one volunteer (13.8 years) for the original data set and undersampled images for AF= [4, 6, 8].

Generally, the reduction of available data for image reconstruction due to undersampling leads to smoothing of image details and suppression of image noise. These effects are observable in all data sets: With increasing AF, the visible part of the sternum body becomes perceptibly smoothed in images of the clavicles; for hand images, an overlap of the muscle tissue with metacarpal bones can be observed and for high acceleration factors details of single teeth become reduced.

**Fig. 2:**
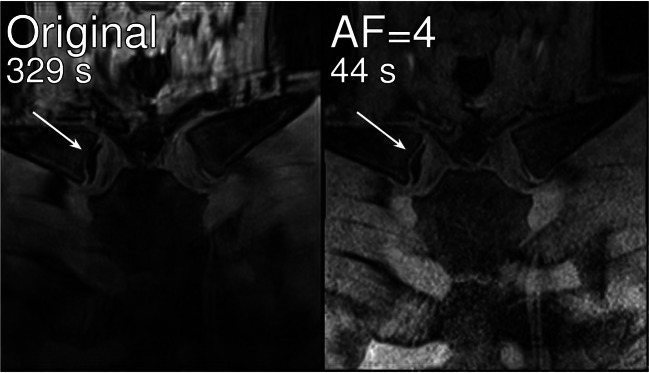
Comparison of an original with an actual accelerated acquisition of the clavicles for one volunteer (17.75 years). Arrows mark an open epiphyseal cartilage clearly visible in the accelerated acquisition but appearing partially ossified in the original scan

Figure 2 shows a comparison of an original image with an actual accelerated acquisition (AF = 4; however, using an acquisition strategy different from the acquisitions described in the Methods section) of the same volunteer (17.75 years) in consecutive scans. The arrow in both images marks an open epiphyseal gap, which appears partially closed in the original image.

### Assessability of reconstructed MR images

Overall assessability for hand images was 100% and roughly 60% (R2: 76%, R3: 70%, R6: 37%) for the clavicles (assessability was higher when the second assessment of the original images is taken into account; R2: 77%, R3: 75%). For teeth images, assessability was around 90% (R4: 91%, R5: 86%; see Table 3 for details). The automated method took all images into account for age estimation.

**Table 3:** Assessability for all data sets using radiological evaluation. Parentheses mark results from a second evaluation

Rater	Position					
**Hand**						
		All	Orig	AF = 4	AF = 8	
R1		96/96	32/32	32/32	32/32	
R3		96/96	32/32	32/32	32/32	
**Clavicles**						
		All	Orig	AF = 2	AF = 3	AF = 4
R2	Left	102(103)/136	31(32)/34	24/34	23/34	24/34
	Right	105(106)/136	32(33)/34	26/34	23/34	24/34
R3	Left	95(102)/136	25(32)/34	22/34	23/34	25/34
	Right	95(102)/136	25(32)/34	23/34	21/34	26/34
R6	left	49/136	19/34	15/34	8/34	7/34
	right	52/136	20/34	14/34	8/34	10/34
**Teeth**						
		All	Orig	AF = 2	AF = 4	AF = 6
R4	Min, 28	124/132	30/33	33/33	31/33	30/33
	Min, 38	124/132	31/33	31/33	31/33	31/33
	Min, 18	116/132	32/33	29/33	28/33	27/33
	Min, 48	117/132	30/33	30/33	29/33	28/33
R5	Min, 28	123/132	31/33	32/33	30/33	30/33
	Min, 38	113/132	29/33	30/33	28/33	26/33
	Min, 18	110/132	29/33	29/33	27/33	25/33
	Min, 48	110/132	29/33	30/33	28/33	23/33

*Orig* original acquisitions, *AF* acceleration factor, *Min* mineralisation

### Reliability of ratings

**Fig. 3:**
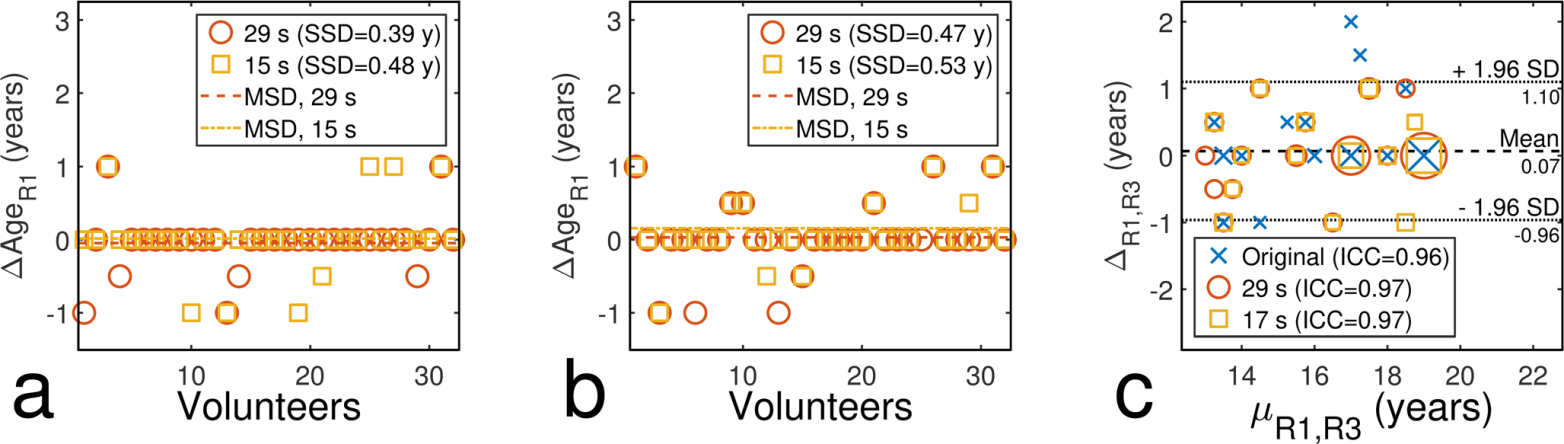
Difference in age estimates of undersampled hand images compared to estimates based on original images for **a** R1 and **b** R3. **c** Bland-Altman plot comparing age estimates of both raters (larger markers denote multiple data points at the same position)

As an exemplary visualisation for all evaluations, Fig. 3 shows the results of the radiological assessment of hand images by raters R1 and R3. Figure 3a, b shows differences to original age estimates separately for both raters and Fig. 3c shows a Bland-Altman plot comparing estimates of both raters (see Table 4 for all results).

**Table 4:** Reliability for all data sets using radiological evaluation

Rater/Position	ICC				SSD (years/stage)			MSD (year/stage)			LOA (year/stage)	*μ*_*BA*_ (year/stage)
**Hand**												
	Orig	AF = 4	AF = 8		AF = 4	AF = 8		AF = 4	AF = 8			
R1	–	0.98	0.97		0.39	0.48		− 0.05	0.02		–	–
R3	–	0.96	0.98		0.57	0.46		− 0.10	0.00		–	–
R1 vs R3	0.93	0.97	0.97		–	–		–	–		0.51	0.05
**Clavicles**												
	Orig	AF = 2	AF = 3	AF = 4	AF = 2	AF = 3	AF = 4	AF = 2	AF = 3	AF = 4		
R2												
Left	0.56	0.69	0.79	0.88	0.81	0.63	0.44	− 0.03	− 0.09	0.26	–	–
Right	0.61	0.71	0.74	0.77	0.78	0.74	0.74	0.04	− 0.04	0.13	–	–
R3												
Left	0.02	0.90	0.05	0.47	0.26	0.73	0.75	0.14	− 0.17	− 0.16	–	–
Right	0.20	0.71	− 0.00	0.52	0.53	0.80	0.73	0.20	− 0.10	− 0.15	–	–
R6												
Left	–	0.97	1.00	0.70	0.22	0.00	0.27	0.09	0.00	0.33	–	–
Right	–	0.92	0.96	0.89	0.46	0.32	0.13	− 0.06	− 0.13	0.07	–	–
R2 vs. R3												
Left	0.27	− 0.42	0.75	0.31	–	–	–	–	–	–	2.00	− 0.17
Right	0.32	− 0.02	0.73	0.65	–	–	–	–	–	–	1.91	− 0.29
R2 vs. R6												
Left	0.62	0.70	0.99	0.59	–	–	–	–	–	–	1.66	0.14
Right	0.95	0.73	0.99	0.94	–	–	–	–	–	–	1.08	− 0.05
R3 vs. R6												
Left	− 0.48	− 0.15	0.91	0.61	–	–	–	–	–	–	2.27	0.40
Right	− 0.38	0.13	0.89	0.68	–	–	–	–	–	–	2.25	0.29
**Teeth**												
	Orig	AF = 2	AF = 4	AF = 6	AF = 2	AF = 4	AF = 6	AF = 2	AF = 4	AF = 6		
R4												
Min, 28	–	0.81	0.93	0.86	0.87	0.57	0.79	0.27	− 0.03	− 0.11	–	–
Min, 38	–	0.79	0.82	0.81	0.85	0.79	0.78	− 0.20	− 0.17	− 0.27	–	–
Min, 18	–	0.80	0.82	0.81	0.98	0.96	0.94	0.07	0.04	− 0.04	–	–
Min, 48	–	0.87	0.86	0.91	0.63	0.69	0.59	0.11	0.11	0.12	–	–
R5												
Min, 28	–	0.97	0.94	0.97	0.37	0.53	0.38	0.00	0.07	0.00	–	–
Min, 38	–	0.98	0.98	0.98	0.31	0.27	0.35	0.10	0.07	0.04	–	–
Min, 18	–	0.98	0.95	0.93	0.33	0.46	0.58	0.04	0.15	0.00	–	–
Min, 48	–	0.94	0.93	0.95	0.50	0.54	0.46	− 0.03	− 0.07	− 0.13	–	–
R4 vs. R5												
Min, 28	0.77	0.87	0.84	0.75	–	–	–	–	–	–	1.65	− 0.38
Min, 38	0.85	0.89	0.94	0.93	–	–	–	–	–	–	1.21	− 0.13
Min, 18	0.67	0.92	0.91	0.86	–	–	–	–	–	–	1.66	− 0.28
Min, 48	0.85	0.88	0.89	0.93	–	–	–	–	–	–	1.20	− 0.17

*Orig* original acquisitions, *AF* acceleration factor, *Min* mineralisation, *LOA* Bland-Altman limits of agreement, *μ*_BA_ Bland-Altman mean

**Fig. 4:**
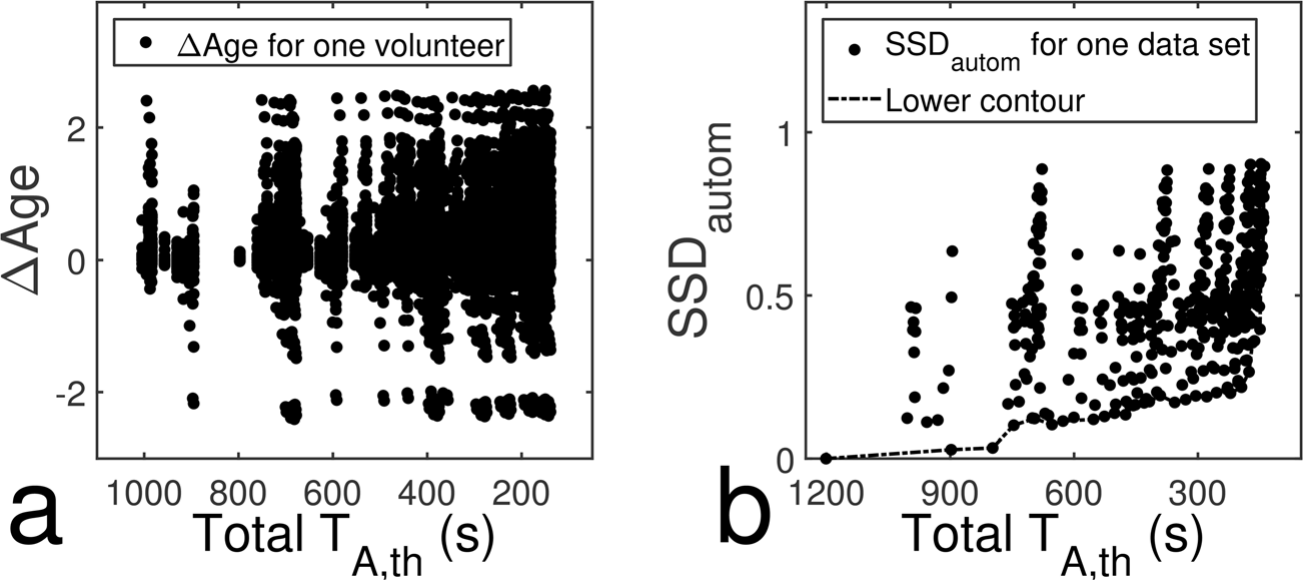
Results of automatic age estimation. **a** ΔAge for all volunteers and data set combinations and **b** SSD for all data set combinations over total theoretical acquisition time. The lower contour marks best-performing combinations

**Fig. 5:**
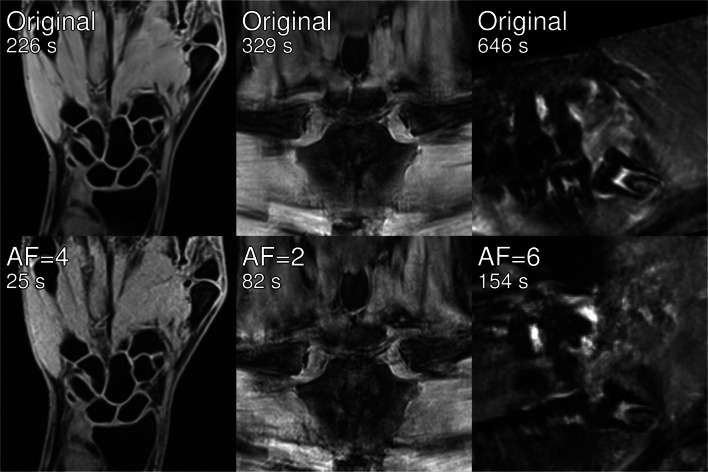
Comparison of original images and actual accelerated acquisitions with a total acquisition time of roughly 4 min. Note, that the real acquisition times differ slightly from the theoretical acquisition times due to scanner restrictions

With the automatic age estimation method, we evaluated the reliability of 441 different data set combinations. A scatter plot showing all differences to original estimates versus total theoretical acquisition time is shown in Fig. 4a. In Fig. 4b, values of SSD of each of the 441 combinations is plotted versus total *T*_A,th_ showing that SSD values were below 0.90 for all combinations. The lower contour of this scatter plot marks data set combinations providing minimum SSD for shortest possible theoretical acquisition times. All combinations of the lower contour yielding SSDs of a maximum of 0.2 years are summarized in Table 5. Regarding agreement with chronological age, the automatic method yielded SSD/MSD = 0.88 years/0.19 years for original images and SSD/MSD = 0.94 years/0.40 years for the last entry in Table 5. Figure 5 shows a comparison of original images and undersampled images acquired with acceleration factors of 4, 2 and 6 for the hand, the clavicles and the teeth, respectively, leading to a total acquisition time of roughly four minutes.

**Table 5:** Best-performing combinations of acceleration factors using the automatic age estimation method

AF_Hand_	AF_Clavicle_	AF_Teeth_	*T*_A,th_ (s)	SSD_autom_ (years)
1	1	2	898	0.03
1	1	3	797	0.03
1	1	4	746	0.10
1	1	6	696	0.12
1	2	2	654	0.10
1	3	2	627	0.11
1	2	3	553	0.12
1	3	3	526	0.13
1	3	4	475	0.13
4	2	2	457	0.16
1	2	6	452	0.16
4	3	2	430	0.17
1	2	9	418	0.17
1	3	9	391	0.19
4	2	3	356	0.17
4	3	3	330	0.18
4	2	4	305	0.19
4	3	4	279	0.19
4	2	6	255	0.20
4	2	9	221	0.20

*AF* acceleration factor, *T*_A,th_ theoretical acquisition time, *SSD*_*autom*_ standard deviation of signed differences for automatic age estimation

## Discussion

In this study, we analysed the reliability of multi-factorial age estimation based on undersampled MRI of all three regions as recommended by the work group for forensic age diagnostics [4]. Radiological analyses showed that a reduction to a total theoretical acquisition time between 4 and 5 min was feasible; the automatic method applied in this study provided reliable results for even shorter acquisition times. This is valuable information since comparable MRI-based age estimation studies use acquisition times of 6 min [15, 38] for the hand and 4 [20] to 6 min [5] for the clavicles. MRI systems with low field strengths can benefit from the fact that lower field strengths lead to shorter values of T_1_, which in turn permit shorter repetition times T_R_ required for the acquisition of one image line. This allows to reduce acquisition time compared to high-field MRI scanners; however, even for such systems, reported acquisition times for hand MRI still range between 1:40 min [13] and 2:44 min [14] leaving room for optimisation. Additionally, the automatic analysis allowed to derive a list of acceleration options providing best reliability for a given amount of time using an entirely objective method evaluating the images’ suitability for age estimation. Our approach can easily be adopted, since CAIPIRINHA is available on current MR scanners and the reconstruction software used in this study is freely available.

The automatic analysis’ results provide deviations of the estimated age with regard to acceleration, which enables a direct evaluation of acceleration limits. For radiological age estimation, the determination of the minimum acceptable theoretical acquisition time requires a more elaborate analysis: The 100% assessability of the images of the hand could be expected due to existing work [27]. Assessability of the teeth was high with no obvious influence of the acceleration factor; however, it was slightly lower for the highest acceleration factor (AF = 6), which may therefore represent an acceleration limit. Correlation was particularly high for the hand; the assessment of the teeth yielded SSD values below 1 stage for both raters and all acceleration factors and MSD values showed no systematic bias. Therefore, the minimum *T*_A,th_ for hand (16 s) and teeth (141 s) can be assumed as guiding values for a lower limit of applicable acquisition times.

Taking the second assessment of the original clavicle images into account, two evaluators—R2 and R3—scored comparable assessability (~ 75%) with decreased assessability for undersampled images but only a small influence of the applied acceleration factor. This could suggest that undersampling in general diminishes assessability. However, this can be explained by the fact that retrospective undersampling is a valid technical approach but the reconstructed data still includes all artefacts (motion, breathing) from the original, long acquisition. Actual undersampled acquisitions show the potential to provide increased image quality compared to long acquisitions or retrospectively undersampled data. This is shown in Fig. 2, where an open epiphyseal gap appears as partially closed in the original image. Evaluator R6 achieved a low overall assessability but also very high reliability for the assessed images. This suggests that quality standards may vary strongly for different raters.

The clavicles are reported to generally be subject to large error ranges [20]. This can also be seen in the intra-rater correlation for the original images, which lies below values reported in the literature [25]. It is known, however, that early and late stages can be confused, which has led to approaches using additional sub-stages [15, 39, 40], or combining or discarding early and late stages [29, 41]. In the current study, no guidelines for unclear cases were defined beforehand, which—in combination with the relatively small subject number—may have led to exaggerated low intra- and inter-rater agreement. Despite the difficulties involved in the analysis of this body region, an acceleration factor of 2 (*T*_A,th_ = 85 s) led to high ICC values and to SSD values well below 1 stage for the clavicles. We, therefore, assume this moderate acceleration as applicable. This leads to a total minimum theoretical acquisition time of roughly 4 min for radiological evaluation. The combination of acceleration factors 4, 2 and 6 for the hand, the clavicles and the teeth, respectively (*T*_A,th_ = 4:15 min) additionally represents a combination that provides reliable results for radiological age estimation and is one of the best-performing combinations of the automatic method (see Table 5). The applicability of these acceleration factors could also be shown in actual accelerated acquisitions.

It is an interesting result that the automatic age estimation method was applied to undersampled data in its original state without additional training. The method provided low overall SSD values and the combinations in Table 5 are well below the uncertainty limit of the radiological methods of 0.5–2 years [42]. The reliability is also shown in the agreement with chronological age, which changed only slightly between the age estimated from original images and the age based on images acquired with a total *T*_A,th_ of 221 s. Furthermore, Table 5 confirms the moderate acceleration potential for acquisitions of the clavicles as indicated by the radiological analysis. This additionally becomes visible in Fig. 4a, where increasing undersampling of the clavicle data leads to repeating high values of ΔAge.

A limitation of this study is the relatively small sample size. This is owed to the fact that the measurements’ raw data were not stored at an earlier stage of our ongoing multi-factorial age estimation study. However, using the concept of systematically increasing the degree of undersampling, the feasibility of our approach could already be shown for the sample size used in this study. We could also see that actual fast acquisitions may provide images with increased quality compared to our original acquisitions; therefore, a study collecting real undersampled data will be considered for future work. It should, however, be noted that the total theoretical acquisition time between 4 and 5 min which was found to still allow reliable age estimation only covers the net acquisition time of the three sequences. An actual examination will require additional time for patient and coil positioning and sequence planning; however, using adequate coil combinations, a repositioning between acquisitions of the clavicles and the teeth may also be replaced by a table move. Furthermore, the automatic method used the *most likely age* approach for the determination of acceleration limits. We believe that the determined optimisation of the acquisition time will also be applicable to studies implementing the *minimum age* concept, which would require to replace the regression analysis by a classification problem; however, we did not investigate this aspect in this study.

This study was performed on a 3T system, which may not always be available. However, we expect our approach to be applicable to lower field strengths, since the feasibility of acceleration techniques could already be shown for field strengths as low as 0.3T [26]. Furthermore, the combination of CAIPIRINHA for image acquisition and TGV for image reconstruction represents a state-of-the-art approach as well as an optimised strategy: CAIPIRINHA modifies the appearance of undersampling artefacts leading to improved image quality and the TGV-based algorithm falls into the class of compressed sensing reconstruction, combining the benefits of both parallel imaging and compressed sensing.

In conclusion, we could show in this study that the total acquisition time for multi-factorial age estimation based on MR images of hand, wisdom teeth and the clavicles can theoretically be reduced to as low as four minutes while still allowing for reliable age estimation.

## Data Availability

The acquired MRI data sets generated and/or analysed during the current study are not publicly available for data privacy reasons. The participants did not explicitly give their consent to freely distribute their imaging data, albeit anonymized.

## Abbreviations

AF: acceleration factor
CAIPIRINHA: Controlled aliasing in parallel imaging results in higher acceleration
FA: Flip angle
GP (method): Greulich and Pyle (age estimation method)
μ_BA_: Bland-Altman mean.
LOA: (Bland-Altman) limits of agreement
MSD: Mean of signed differences
SSD: Standard deviation of signed differences
*T*_A_: Acquisition time
*T*_A,th_: Theoretical acquisition time (based on retrospective undersampling)
TF: Turbo factor
TSE: Turbo spin-echo
TGV: Total generalized variation
VIBE: Volumetric interpolated breath hold examination

## References

[1] Martin DD, Wit JM, Hochberg Z, Sävendahl L, van Rijn R, Fricke O, Cameron N, Caliebe J, Hertel T, Kiepe D, Albertsson-Wikland K, Thodberg HH, Binder G, Ranke MB (2011). The use of bone age in clinical practice—part 1. Horm Res Paediatr.

[2] Lee SC, Shim JS, Seo SW, Lim KS, Ko KR (2013). The accuracy of current methods in determining the timing of epiphysiodesis. Bone Jt J.

[3] Latham KE, Bartelink EJ, Finnegan M (2018) New perspectives in forensic human skeletal identification. Academic Press

[4] Schmeling A, Garamendi MP, Prieto JL, Landa IM (2011) Forensic age estimation in unaccompanied minors and young living adults. In: Vieira DN (ed) Forensic medicine - from old problems to new challenges. InTech, pp 77–120

[5] Vieth V, Schulz R, Brinkmeier P, Dvorak J, Schmeling A (2014). Age estimation in U-20 football players using 3.0 tesla MRI of the clavicle. Forensic Sci Intl.

[6] Timme M, Steinacker JM, Schmeling A (2017). Age estimation in competitive sports. Int J Legal Med.

[7] Cameron N (2015). Can maturity indicators be used to estimate chronological age in children?. Ann Hum Biol.

[8] Liversidge HM, Buckberry J, Marquez-Grant N (2015). Age estimation. Ann Hum Biol.

[9] Greulich W, Pyle S (1959) Radiographic atlas of skeletal development of the hand and wrist. Combined Academic Publ

[10] Tanner JM, Whitehouse RH, Cameron N, Marshall WA, Healy MJR (1983) Assessment of skeletal maturity and prediction of adult height (TW2 method). Academic Press

[11] Demirjian A, Goldstein H, Tanner JM (1973). A new system of dental age assessment. Hum Biol.

[12] Schulz R, Mühler M, Mutze S, Schmidt S, Reisinger W, Schmeling A (2005). Studies on the time frame for ossification of the medial epiphysis of the clavicle as revealed by CT scans. Int J Legal Med.

[13] Serinelli S, Panebianco V, Martino M, Battisti S, Rodacki K, Marinelli E, Zaccagna F, Semelka RC, Tomei E (2015). Accuracy of MRI skeletal age estimation for subjects 12--19. Potential use for subjects of unknown age. Int J Legal Med.

[14] Terada Y, Kono S, Tamada D, Uchiumi T, Kose K, Miyagi R, Yamabe E, Yoshioka H (2013). Skeletal age assessment in children using an open compact MRI system. Magn Reson Med.

[15] De Tobel J, Hillewig E, de Haas MB (2019). Forensic age estimation based on T1 SE and VIBE wrist MRI: do a one-fits-all staging technique and age estimation model apply?. Eur Radiol.

[16] De Tobel J, Hillewig E, Verstraete K (2017). Forensic age estimation based on magnetic resonance imaging of third molars: converting 2D staging into 3D staging. Ann Hum Biol.

[17] Baumann P, Widek T, Merkens H (2015). Dental age estimation of living persons: comparison of MRI with OPG. Forensic Sci Intl.

[18] Widek T, Genet P, Merkens H, Boldt J, Petrovic A, Vallis J, Scheurer E (2019). Dental age estimation: the chronology of mineralization and eruption of male third molars with 3T MRI. Forensic Sci Int.

[19] Schmidt S, Ottow C, Pfeiffer H, Heindel W, Vieth V, Schmeling A, Schulz R (2017). Magnetic resonance imaging-based evaluation of ossification of the medial clavicular epiphysis in forensic age assessment. Int J Legal Med.

[20] Hillewig E, Degroote J, Van Der Paelt T (2013). Magnetic resonance imaging of the sternal extremity of the clavicle in forensic age estimation: towards more sound age estimates. Int J Legal Med.

[21] Tangmose S, Jensen KE, Villa C, Lynnerup N (2014). Forensic age estimation from the clavicle using 1.0T MRI—preliminary results. Forensic Sci Int.

[22] Martínez Vera NP, Höller J, Widek T, Neumayer B, Ehammer T, Urschler M (2017). Forensic age estimation by morphometric analysis of the manubrium from 3D MR images. Forensic Sci Int.

[23] Ottow C, Schulz R, Pfeiffer H, Heindel W, Schmeling A, Vieth V (2017). Forensic age estimation by magnetic resonance imaging of the knee: the definite relevance in bony fusion of the distal femoral- and the proximal tibial epiphyses using closest-to-bone T1 TSE sequence. Eur Radiol.

[24] Vieth V, Schulz R, Heindel W, Pfeiffer H, Buerke B, Schmeling A, Ottow C (2018). Forensic age assessment by 3.0T MRI of the knee: proposal of a new MRI classification of ossification stages. Eur Radiol.

[25] Hillewig E, De Tobel J, Cuche O, Vandemaele P, Piette M, Verstraete K (2011). Magnetic resonance imaging of the medial extremity of the clavicle in forensic bone age determination: a new four-minute approach. Eur Radiol.

[26] Terada Y, Tamada D, Kose K (2015). Acceleration of skeletal age MR examination using compressed sensing. J Magn Reson Imaging.

[27] Neumayer B, Schloegl M, Payer C (2018). Reducing acquisition time for MRI-based forensic age estimation. Sci Rep.

[28] Štern D, Payer C, Giuliani N, Urschler M (2018). Automatic age estimation and majority age classification from multi-factorial MRI data. IEEE J Biomed Health Inform.

[29] De Tobel J (2019) Multi-factorial forensic age estimation: combining magnetic resonance imaging of the third molars, the left wrist and both clavicles. Ghent University. Faculty of Medicine and Health Sciences ; KU Leuven. Doctoral School Biomedical Sciences

[30] Pruessmann KP, Weiger M, Scheidegger MB, Boesiger P (1999). SENSE: sensitivity encoding for fast MRI. Magn Reson Med.

[31] Griswold MA, Jakob PM, Heidemann RM, Nittka M, Jellus V, Wang J, Kiefer B, Haase A (2002). Generalized autocalibrating partially parallel acquisitions (GRAPPA). Magn Reson Med.

[32] Lustig M, Donoho DL, Pauly JM (2007). Sparse MRI: the application of compressed sensing for rapid MR imaging. Magn Reson Med.

[33] Breuer FA, Blaimer M, Heidemann RM, Mueller MF, Griswold MA, Jakob PM (2005). Controlled aliasing in parallel imaging results in higher acceleration (CAIPIRINHA) for multi-slice imaging. Magn Reson Med.

[34] Schloegl M, Holler M, Schwarzl A, Bredies K, Stollberger R (2017). Infimal convolution of total generalized variation functionals for dynamic MRI. Magn Reson Med.

[35] Uecker M, Lai P, Murphy MJ, Virtue P, Elad M, Pauly JM, Vasanawala SS, Lustig M (2014). ESPIRiT—an eigenvalue approach to autocalibrating parallel MRI: where SENSE meets GRAPPA. Magn Reson Med.

[36] Knoll F, Bredies K, Pock T, Stollberger R (2010). Second order total generalized variation (TGV) for MRI. Magn Reson Med.

[37] Urschler M, Krauskopf A, Widek T (2016). Applicability of Greulich--Pyle and Tanner–Whitehouse grading methods to MRI when assessing hand bone age in forensic age estimation: a pilot study. Forensic Sci Intl.

[38] Schmidt S, Vieth V, Timme M, Dvorak J, Schmeling A (2015). Examination of ossification of the distal radial epiphysis using magnetic resonance imaging. New insights for age estimation in young footballers in FIFA tournaments. Sci Justice.

[39] Kellinghaus M, Schulz R, Vieth V, Schmidt S, Pfeiffer H, Schmeling A (2010). Enhanced possibilities to make statements on the ossification status of the medial clavicular epiphysis using an amplified staging scheme in evaluating thin-slice CT scans. Int J Legal Med.

[40] Wittschieber D, Schmidt S, Vieth V (2014). Subclassification of clavicular substage 3a is useful for diagnosing the age of 17 years. Rechtsmedizin.

[41] De Tobel J, Hillewig E, van Wijk M (2019). Staging Clavicular development on.

[42] Ritz-Timme S, Cattaneo C, Collins MJ, Waite ER, Schütz HW, Kaatsch HJ, Borrman HI (2000). Age estimation: the state of the art in relation to the specific demands of forensic practise. Int J Legal Med.

